# Clinical Reasoning and Diagnostic Challenge in a 23-Year-Old Man With Rapidly Progressive Dysphagia and Hypophonia: Juvenile-Onset Amyotrophic Lateral Sclerosis Caused by a FUS Gene Mutation

**DOI:** 10.7759/cureus.93369

**Published:** 2025-09-27

**Authors:** Zhuo Luan, Isabel V Narvaez-Correa, Jithendhar Kandimalla, Ramon G Valles, Paisith Piriyawat

**Affiliations:** 1 Neurology, Texas Tech University Health Sciences Center El Paso, El Paso, USA; 2 Internal Medicine, Texas Tech University Health Sciences Center El Paso, El Paso, USA

**Keywords:** fus mutations, genetic test, hypophonia, progressive dysphagia, young-onset als

## Abstract

Dysphagia and dysphonia of unclear etiology in young adults pose a significant diagnostic challenge, as these symptoms are more commonly attributed to benign or structural causes rather than serious neurodegenerative disease. The absence of classic neuromuscular signs such as limb weakness, hyperreflexia, or fasciculations can delay consideration of motor neuron disease, particularly when bulbar symptoms occur in isolation. We describe a previously healthy 23-year-old man who presented with rapidly progressive dysphagia and hypophonia, initially misattributed to infectious causes. Despite an extensive workup for structural, autoimmune, and infectious causes, no clear etiology was identified. Neurologic examination revealed predominantly bulbar dysfunction, and electrodiagnostic studies showed acute to subacute denervation changes in the tongue and trapezius muscles. Genetic testing confirmed juvenile-onset amyotrophic lateral sclerosis due to a pathogenic FUS gene mutation (p.Pro525Leu). This case highlights the importance of including motor neuron disease in the differential diagnosis of rapidly progressive bulbar symptoms of unknown origin. It highlights the importance of early electrodiagnostic testing and genetic evaluation in establishing a diagnosis.

## Introduction

Dysphagia and dysphonia are frequently encountered symptoms in clinical practice and are typically attributed to benign or self-limited conditions. Common causes include upper respiratory tract infections, laryngitis, gastroesophageal reflux disease, allergic reactions, or mechanical issues such as vocal cord nodules, polyps, or esophageal strictures. In most cases, these symptoms are transient and resolve with conservative treatment [1,2]. However, when symptoms persist, progress, and remain unexplained by standard evaluations, the diagnostic process becomes significantly more complex. The differential diagnosis is broad and includes neoplastic, infectious, autoimmune, structural, and neurologic causes. In young adults, structural and inflammatory causes are often prioritized [3], while more serious neurodegenerative conditions may not be immediately considered, especially when limb weakness or other systemic signs are absent.

From a neurologic perspective, bulbar dysfunction resulting in dysphagia and dysphonia can arise from lesions affecting the brainstem, cranial nerves, neuromuscular junction, or bulbar musculature [4]. Myasthenia gravis (MG), multiple sclerosis, brainstem infarcts, demyelinating disease, motor neuron disease, and inflammatory myopathies such as inclusion body myositis (IBM) or polymyositis are all potential considerations. Each of these conditions has overlapping clinical features, and distinguishing among them can be particularly challenging in the early stages when symptoms are subtle or nonspecific. The diagnostic challenge is further compounded in cases of isolated bulbar involvement, where classic neurologic signs such as limb weakness, upper motor neuron findings, or sensory deficits may be absent or obscured, particularly when the clinical picture is dominated by severe dysphagia and its consequences, such as profound malnutrition.

Juvenile-onset amyotrophic lateral sclerosis (ALS) is a rare and often overlooked cause of progressive bulbar dysfunction in young adults [5]. ALS is a progressive neurodegenerative disorder characterized by the degeneration of upper and lower motor neurons, typically presenting between the ages of 50 and 70. Juvenile-onset cases, defined by symptom onset before the age of 25, are uncommon and often linked to genetic mutations. Among these, mutations in the FUS gene have been associated with a particularly aggressive phenotype characterized by rapid progression, early respiratory failure, and poor prognosis. The FUS P525L mutation, in particular, has been strongly associated with early-onset bulbar-predominant ALS and is known to lead to early tracheostomy and dependence on feeding tubes within the first year of symptom onset [6,7].

Here, we present the case of a previously healthy 23-year-old man who developed rapidly progressive dysphagia and hypophonia that were initially misdiagnosed as infectious in nature. An extensive structural, autoimmune, and infectious workup was unrevealing. Ultimately, electrodiagnostic testing and genetic analysis confirmed the diagnosis of juvenile-onset ALS due to a pathogenic FUS P525L mutation. This case highlights the importance of maintaining a broad differential diagnosis in young patients with bulbar symptoms of unclear etiology. It highlights the crucial role of early recognition, electrodiagnostic testing, and genetic evaluation in achieving a timely diagnosis.

## Case presentation

A 23-year-old man with no past medical history presented to the emergency department with rapidly progressive dysphagia and hypophonia. Four months prior, his symptoms began with the gradual onset of hoarseness and a sore throat. He sought care from his primary care physician and was diagnosed with presumed tonsillitis. A 10-day course of amoxicillin yielded no improvement. A neck ultrasound was performed in the outpatient setting and was unremarkable. Despite treatment, his symptoms continued to progress. His voice gradually declined in volume until it was reduced to a low mumble. Two months prior, he began experiencing frequent choking episodes, particularly with liquids and saliva. One month prior, he developed difficulty swallowing solid food and required significant effort to clear his oropharynx during meals. During this time, he experienced profound unintentional weight loss of approximately 60 kg over four months, likely related to decreased oral intake. He was noted to be extremely underweight with a BMI of 12. According to his family, he had not experienced ptosis, diplopia, facial droop, limb weakness, paresthesia, or gait instability. He remained fully ambulatory until the day of presentation. On the day of admission, while awaiting evaluation, he experienced a seizure-like episode characterized by eye-rolling, unresponsiveness, and tonic-clonic movements of the lower extremities. He was found to be hypotensive, hypopneic, intermittently responsive, and unable to protect his airway. He was emergently intubated for airway protection and admitted to the intensive care unit. Levetiracetam 500 mg twice daily was initiated for seizure prophylaxis. EEG demonstrated moderate encephalopathy, suggesting that the seizures may have been provoked by rapidly developing malnutrition or metabolic/electrolyte disturbances, similar to those seen in anorexia nervosa. A complete neurologic examination could not be performed until the patient was extubated three days later.

His past medical history was unremarkable. He had no history of smoking, alcohol use, or illicit drug use. There was no known personal or family history of autoimmune, neuromuscular, or metabolic disorders. However, his family history was notable for his biological mother, who reportedly developed dysphagia in her early 20s during pregnancy and died two months postpartum. No formal diagnosis was documented, but her symptoms were described as similar in character and progression.

A full neurologic examination was performed on the third day of admission following extubation. The patient was awake and alert. Cranial nerve examination revealed symmetric pupils with intact extraocular movements, no nystagmus, and no ptosis. Sensation to light touch was intact over the face bilaterally. The face appeared symmetric, although there was bilateral weakness of cheek puff and eye closure. Tongue movements were significantly weak, with notable atrophy. Motor examination demonstrated mild to moderate symmetric weakness. Strength in the upper extremities ranged from 3+ to 4+/5, with the most significant weakness observed in arm abduction. Lower extremity strength ranged from 3+ to 5/5, with the most significant weakness in hip flexion. Sensory examination was intact to light touch, temperature, pinprick, and vibration. Deep tendon reflexes were asymmetric, with diminished responses in the upper extremities and preserved or brisk responses in the lower extremities. Specifically, the biceps reflex was absent on the right (0+) and reduced on the left (1+); triceps reflexes were similarly absent on the right (0+) and reduced on the left (1+); and brachioradialis reflexes were absent on the right (0+) and reduced on the left (1+). In contrast, patellar reflexes were brisk and symmetric at 3+ bilaterally, while Achilles reflexes were normal at 2+ bilaterally. Plantar responses were downgoing bilaterally, and Hoffmann signs were negative on both sides.

Initial concerns included the possibility of an intracranial lesion or esophageal obstruction. A stat non-contrast CT of the head and CTA of the head and neck revealed no acute findings, including no large vessel occlusion or intracranial hemorrhage. CTA of the chest and contrast-enhanced CT of the thorax, abdomen, and pelvis did not reveal any neck or gastrointestinal lesions or tumors. The only notable finding was severe constipation at the level of the rectum. An esophagogastroduodenoscopy was performed to rule out structural abnormalities of the esophagus and showed no evidence of any esophageal lesions. The patient demonstrated prominent, progressive bulbar palsy characterized by tongue weakness and atrophy, mild to moderate symmetric limb weakness, and markedly diminished upper extremity reflexes with preserved lower extremity reflexes and intact sensation. These findings could be suggestive of motor neuron pathology. However, the examination may not fully reflect the underlying pathology due to the patient’s severe deconditioning and recent intubation from critical illness. Some observed deficits may be secondary to generalized weakness rather than primary neurologic dysfunction. Even after extubation, a formal swallow evaluation could not be performed because the patient remained unable to swallow, making it unclear which phase of swallowing (oral, pharyngeal, or esophageal) was most affected. While motor neuron disease remained high on the differential, the lesion could also involve the brainstem, particularly the cranial nerve nuclei, such as the glossopharyngeal or hypoglossal nerves, or could localize to the peripheral nerves, neuromuscular junction, or even the muscles. Consequently, alternative diagnoses, including brainstem stroke, tumor, sarcoidosis, central or peripheral demyelination, MG, Chagas disease, oculopharyngeal muscular dystrophy (OPMD), IBM, or paraneoplastic bulbar syndrome, could not be definitively ruled out at this stage.

To further investigate these possibilities, a comprehensive diagnostic workup was undertaken to evaluate structural, inflammatory, infectious, autoimmune, and paraneoplastic etiologies. Pan-CT showed no evidence of tumors. Brain MRI with and without contrast was performed to assess for brainstem stroke, demyelinating disease, or mass lesions involving cranial nerve nuclei, particularly the glossopharyngeal and hypoglossal nuclei or cranial nerves, but revealed no acute infarction, mass effect, or radiographic evidence of demyelination or neoplasm. Given the bulbar presentation, MG was considered. Although uncommon, MG can present with neurogenic tongue atrophy, especially in longstanding, severe, or bulbar-predominant cases [8-10], so this possibility should not be overlooked during evaluation. However, the patient lacked hallmark fluctuating symptoms typically associated with MG, such as diplopia, ptosis, or diurnal variation in weakness. Furthermore, a comprehensive antibody panel including acetylcholine receptor binding, blocking, and modulating antibodies, as well as muscle-specific kinase (MuSK) antibodies, was negative, making seropositive MG less likely (Table 1). Although bulbar-predominant MG can be seronegative, the absence of classic fluctuating symptoms and upper motor neuron signs such as brisk lower extremity reflexes argues against MG as the primary diagnosis. Nonetheless, further electrophysiological studies, such as repetitive nerve stimulation, were warranted to exclude seronegative MG.

**Table 1:** Serological lab work The serologic and infectious workup was unremarkable, with normal thyroid function, negative acetylcholine receptor and MuSK antibodies, non-reactive *Trypanosoma cruzi* and HIV serologies, and negative blood cultures. TSH: thyroid-stimulating hormone, CK: creatine kinase, rec: receptor, Ab: antibody, MUSK: muscle-specific kinase, HIV: human immunodeficiency virus

Serum	Value	Reference
TSH	2.660	0.465-4.68 MIU/L
CK	59	55-170 IU/L
Acetylcholine rec bind Ab	<0.30	*≤*0.30 nmol/L
Acetylcholine rec bloc Ab	<15%	<15% inhibition
Acetylcholine rec mod Ab	<1%	<32% inhibition
Anti-MUSK Ab	<1:10	<1:10
*Trypanosoma cruzi* Ab, total	Nonreactive	Nonreactive
HIV ½ antibodies	Negative	Negative
Blood culture	Negative	Negative

IBM was also considered, particularly given the patient’s progressive dysphagia [11]. However, several features made this diagnosis unlikely. IBM typically presents with a characteristic pattern of slowly progressive, asymmetric weakness affecting the finger flexors and quadriceps, which was not observed in this patient. Neurological examination did not reveal focal distal weakness, and there was no evidence of muscle tenderness, atrophy limited to distal muscles, or percussion myotonia. Additionally, creatine kinase levels were within normal limits (Table 1), which, although not definitively excluding IBM, suggests that an active inflammatory myopathy is unlikely. Importantly, the typical age of onset for IBM is over 50 years old, whereas this patient is in his early 20s, making the diagnosis highly improbable. Similarly, OPMD, though associated with progressive dysphagia, was considered unlikely in this case. OPMD classically presents in mid-to-late adulthood, typically after the age of 40, with slowly progressive ptosis and dysphagia [12].

In contrast, this patient is in his early 20s and exhibited no eyelid ptosis. Moreover, the rapid progression of symptoms over just a few months is atypical for OPMD, which usually evolves over years. Nonetheless, electromyography (EMG) may still be considered to help rule out OPMD, as it can detect myopathic changes in affected muscles, particularly in the cricopharyngeal, facial, and proximal limb muscles, which may support or argue against a myopathic etiology if uncertainty remains. Infectious causes such as Chagas disease were considered, given the potential for neuromuscular involvement. However, the patient denied any recent or remote travel to endemic areas, and serologic testing for *Trypanosoma cruzi* antibodies was negative (Table 1), ruling out Chagas disease as a cause of neuromuscular dysfunction. Other serologic and infectious workups were unrevealing. Tests, including HIV-1/2 antibodies, thyroid-stimulating hormone, rapid and 24-hour streptococcal screens, as well as blood, urine, and respiratory cultures, were all negative (Table 1). Additionally, the hepatitis panel and multiplex respiratory pathogen panel (BioFire) returned negative, ruling out common viral and bacterial etiologies.

Alternative diagnoses were thoroughly considered; however, the constellation of prominent bulbar symptoms, tongue atrophy, and preserved sensation without radiographic or serologic evidence of structural, infectious, or autoimmune disease strongly favored diffuse motor neuron pathology, raising concern for motor neuron disease as the leading etiology. Therefore, inpatient nerve conduction velocity (NCV) and EMG studies were pursued, as these are the standard diagnostic tests used to evaluate for motor neuron disease and to further characterize the pattern of neuromuscular involvement.

Nerve conduction studies revealed normal sensory responses in the bilateral superficial peroneal, right sural, right median, and right ulnar nerves (Table 2). Motor conduction studies showed normal distal latencies, amplitudes, and conduction velocities in the right median, right ulnar, and bilateral tibial nerves. The bilateral peroneal nerves demonstrated mild conduction slowing between the right fibular head and popliteal fossa but were otherwise unremarkable (Table 3). F-wave latencies were normal in the right ulnar and tibial nerves but absent in the right median, bilateral peroneal, and left tibial nerves (Table 4). Repetitive nerve stimulation of the right facial and ulnar nerves was limited by movement artifacts; intermittent decrements greater than 10% were observed but were inconsistent and lacked the characteristic U-shaped pattern typical of neuromuscular junction disorders (Table 5). Needle EMG revealed abnormal spontaneous activity, including fibrillation potentials and positive sharp waves, in the right tongue and bilateral trapezius muscles. Reduced recruitment was noted in most muscles tested. Polyphasic motor units with normal amplitudes were seen in multiple limb and truncal muscles, while the left gastrocnemius showed large-amplitude motor units (Table 6). In summary, the electrodiagnostic findings demonstrated acute to subacute denervation changes in the tongue and trapezius muscles, raising concern for motor neuron pathology. Since the study was limited by movement artifacts during repetitive nerve stimulation, neuromuscular junction pathology like MG could not be definitively ruled out.

**Table 2:** Sensory nerve conduction study The study showed that normal sensory responses were present in the bilateral superficial peroneal, right sural, right median, and right ulnar nerves. Rec. site: recording site, onset lat: onset latency, peak lat: peak latency, NP amp: negative peak amplitude, PP amp: peak-to-peak amplitude, onset dif: onset latency difference, peak dif: peak latency difference, onset vel: onset conduction velocity, R: right, L: left, dig II: digit II, dig V: digit V, lat leg: lateral leg

Nerve/sites	Rec. site	Onset lat (ms)	Peak lat (ms)	NP amp (µV)	PP amp (µV)	Distance (cm)	Onset dif (ms)	Peak dif (ms)	Onset vel (m/s)
R median - dig II (antidromic)									
Wrist	Index	2.3	3.1	33.3	40.6	14	2.3	3.1	59.7
R ulnar - dig V (antidromic)									
Wrist	Dig V	2.1	2.8	34.7	29.8	14	2.1	2.8	65.6
R sural (antidromic)									
Calf	Ankle	1.7	2	17.1	24.9	14	1.7	2	84
L superficial peroneal (antidromic)									
Lat leg	Ankle	2.2	2.5	15.4	16.9	14	2.2	2.5	63.4
R superficial peroneal (antidromic)									
Lat leg	Ankle	2	2.4	15.7	16.3	14	2	2.4	68.6

**Table 3:** Motor nerve conduction study The study revealed normal distal latencies, amplitudes, and conduction velocities in the right median, right ulnar, and bilateral tibial nerves. The bilateral peroneal nerves demonstrated mild conduction slowing between the right fibular head and popliteal fossa but were otherwise unremarkable. lat dif: latency difference, dist.: distance, N/A: not applicable, R: right, L: left, APB: abductor pollicis brevis, ADM: abductor digiti minimi, EDB: extensor digitorum brevis, AH: abductor hallucis, A: above, B: below

Nerve/sites	Muscle	Latency (ms)	Amplitude	Segments	Dist. (cm)	Lat dif (ms)	Velocity (m/s)
R median - APB							
Wrist	APB	3.88	6.1	Wrist - APB	8	N/A	N/A
Elbow	APB	7.46	6.6	Elbow - wrist	23	3.58	64.2
R ulnar - ADM							
Wrist	ADM	2.6	8	Wrist - ADM	8	N/A	N/A
B. elbow	ADM	5.67	7.3	B. elbow - wrist	17	3.06	55.5
A. elbow	ADM	7.35	7.4	A. elbow - B. elbow	10	1.69	59.3
L peroneal - EDB							
Ankle	EDB	6.23	2.8	Ankle - EDB	8	N/A	N/A
B. fib head	EDB	13.1	2.8	B. fib head - ankle	30	6.88	43.6
A. fib head	EDB	13.54	7.8	A. fib head - B. fib head	10	0.44	228.6
Tibial-ankle	EDB	3.69	9.3	Tibial-ankle - EDB			
R peroneal - EDB							
Ankle	EDB	3.42	4.4	Ankle - EDB	8	N/A	N/A
B. fib head	EDB	8.79	4.1	B. fib head - ankle	29	5.38	54
A. fib head	EDB	12.08	8.1	A. fib head - B. fib head	10	3.29	30.4
Tibial-ankle	EDB	3.4	6.6	Tibial-ankle - EDB			
L tibial - AH							
Ankle	AH	4.94	11.6	Ankle - AH	8	N/A	N/A
Knee	AH	12.98	9.1	Knee - ankle	36	8.04	44.8
R tibial - AH							
Ankle	AH	6.1	13.8	Ankle - AH	8	N/A	N/A
Knee	AH	13.54	12.6	Knee - ankle	38	7.44	51.1

**Table 4:** F-waves F-wave latencies were typical in the right ulnar and tibial nerves but absent in the right median, bilateral peroneal, and left tibial nerves. R: right, L: left, APB: abductor pollicis brevis, ADM: abductor digiti minimi, EDB: extensor digitorum brevis, AH: abductor hallucis, lat: latency, NR: no response

Nerve	F lat (ms)	M lat (ms)	F-M lat (ms)
R median - APB	NR	NR	NR
R ulnar - ADM	26	3	23
L peroneal - EDB	NR	NR	NR
R peroneal - EDB	NR	NR	NR
L tibial - AH	NR	NR	NR
R tibial - AH	29.7	4.6	25.2

**Table 5:** Repetitive nerve stimulation Repetitive nerve stimulation of the right facial and ulnar nerves was limited by movement artifacts; intermittent decrements greater than 10% were observed but were inconsistent and lacked the characteristic U-shaped pattern typical of neuromuscular junction disorders. ampl: amplitude, R: right, ADM: abductor digiti minimi

Anatomy/train	Ampl (mV)	d. ampl1 (%)	Fac ampl (%)	Area (mVms)	d. area1 (%)	Fac area (%)	Rate (Hz)
R facial - nasalis							
Baseline	0.9	-19.1	100	4.8	-48.3	100	3
Baseline	0.7	35.9	80.8	3.8	-54.7	78.9	3
Post exercise	0.9	6.3	105.2	4.9	-17.5	103.7	3
@ 1:00	1	-2.1	117	5.3	-4.4	110.3	3
@ 2:00	1	4.4	114.5	5.1	0.6	107.1	3
@ 3:00	1.1	-11.3	120.3	5	-15.4	105.1	3
@ 4:00	1	-8.7	119.1	5	-32.5	104.4	3
@ 5:00	1.2	-31.8	140	0	0	0	3
R ulnar - ADM							
Baseline	5.9	20.8	100	14.9	16	100	3
Baseline	6.2	-10	106.1	15.8	-18.2	106	3
@ 1:00	7.2	7.4	122.3	17.7	-18.2	118.8	3
@ 2:00	6.8	12	115.3	16.2	-7.2	109.2	3
@ 3:00	5.9	18.3	100.6	16.3	-18.3	109.7	3
@ 4:00	6.1	15.6	103.7	14.9	-5.6	100.2	3
@ 5:00	6.3	14	107.3	15.5	-4.4	104.5	3

**Table 6:** EMG study The EMG demonstrated acute to subacute denervation changes in the tongue and trapezius muscles, raising concern for motor neuron pathology. EMG: electromyography, MUP: motor unit potential, IA: insertional activity, Fib: fibrillation potentials, PSW: positive sharp waves, Fasc: fasciculations, H.F.: high-frequency discharges, Amp: amplitude, Dur.: duration, PPP: polyphasic potentials, R: right, L: left, N: normal, N/A: not applicable, C: cervical spinal root, L: lumbar spinal root, S: sacral spinal root

		Spontaneous					MUP			Recruitment
Muscle	Nerve	Roots	IA	Fib	PSW	Fasc	H.F.	Amp	Dur.	PPP	Pattern
R. tibialis anterior	Deep peroneal (fibular)	L4-L5	N	None	None	None	None	N	N	N	Reduced
R. gastrocnemius (medial head)	Tibial	S1-S2	N	None	None	None	None	N	N	N	Reduced
R. vastus medialis	Femoral	L2-L4	N	None	None	None	None	N	N	N	Reduced
R. extensor digitorum communis	Radial	C7-C8	N	None	None	None	None	N	N	2+	Reduced
R. first dorsal interosseous	Ulnar	C8-T1	N	None	None	None	None	N	N	1+	Reduced
R. triceps brachii	Radial	C6-C8	N	None	None	None	None	N	N	N	Reduced
L. tibialis anterior	Deep peroneal (fibular)	L4-L5	N	None	None	None	None	N	2+	2+	Reduced
L. gastrocnemius (medial head)	Tibial	S1-S2	N	None	None	None	None	5k	N	N	Reduced
L. vastus medialis	Femoral	L2-L4	N	None	None	None	None	N	N	N	Reduced
L. first dorsal interosseous	Ulnar	C8-T1	N	None	None	None	None	N	N	N	Reduced
L. extensor digitorum communis	Radial	C7-C8	N	None	None	None	None	N	2+	2+	Reduced
L. deltoid	Axillary	C5-C6	1+	None	None	None	None	N	N	2+	Reduced
R. tongue	Hypoglossal	Medulla	3+	2+	None	None	None	N	N/A	N/A	N/A
R. trapezius (upper)	Accessory (spinal)	C3-C4	2+	2+	1+	None	None	N	N	2+	N/A
R. cervical paraspinals	Spinal	C4-C8	N	None	None	None	None	N	N	2+	N/A

Given the NCV/EMG findings, the patient’s young age, and a family history suggestive of similar symptoms in his mother, a hereditary bulbar-onset motor neuron disease was strongly suspected. Genetic testing was performed using the Invitae ALS panel, which screens for mutations in genes associated with ALS. The patient tested negative for the C9orf72 hexanucleotide repeat expansion, with two normal alleles containing two and seven GGGGCC repeats. He was also found to be homozygous for the normal ATXN2 allele, with 22 CAG trinucleotide repeats. Notably, the patient carried a heterozygous pathogenic variant in the FUS gene: c.1574C>T, resulting in the missense mutation p.Pro525Leu (P525L), a well-established mutation associated with juvenile-onset aggressive ALS [5,6].

During hospitalization, the patient developed pneumonia, which necessitated reintubation. Due to persistent dysphagia and inability to protect his airway, he underwent tracheostomy and percutaneous endoscopic gastrostomy (PEG) tube placement on hospital day 7. Levetiracetam, which had been initiated for seizure prophylaxis, was discontinued on hospital day 30, as the seizures were determined to be provoked in the context of dehydration. The patient was started on riluzole but was later transitioned to edaravone (Radicava) due to mild transaminitis. He was discharged home on hospital day 50. At a one-month virtual follow-up, he was ambulatory without assistance. He remains on ventilatory support via tracheostomy and receives enteral nutrition through the PEG tube. A referral for genetic counseling was also provided to the patient.

## Discussion

This case highlights a rare and aggressive form of juvenile-onset ALS caused by a pathogenic FUS gene variant (p.Pro525Leu). The patient, a previously healthy 23-year-old man, initially presented with isolated bulbar symptoms that progressed rapidly over four months, leading to severe dysphagia and associated malnutrition, and eventually PEG tube and trach dependency. The early age of onset and bulbar-predominant presentation posed significant diagnostic challenges, particularly in the setting of profound deconditioning and associated limited physical examination. The differential diagnosis for progressive bulbar dysfunction in a young adult is broad and includes structural, inflammatory, autoimmune, infectious, and neuromuscular disorders. EMG/NCS demonstrated denervation changes in bulbar and proximal limb muscles, raising suspicion for motor neuron disease. Genetic testing ultimately confirmed the diagnosis, identifying a pathogenic heterozygous missense mutation in the FUS gene, c.1574C>T (p.Pro525Leu), a well-established variant associated with juvenile-onset ALS and rapid disease progression [5].

The FUS gene encodes an RNA-binding protein involved in DNA repair and RNA metabolism. The P525L missense mutation disrupts the nuclear localization signal, resulting in cytoplasmic mislocalization and toxic aggregation within motor neurons [13]. Clinically, this variant is associated with one of the most severe forms of ALS. Patients typically present in adolescence or early adulthood with rapidly progressive motor weakness, often beginning with bulbar or proximal limb involvement. Prominent bulbar dysfunction, including dysarthria, dysphagia, and tongue atrophy, develops early and is accompanied by symmetric limb weakness, brisk reflexes, and preserved sensation. Respiratory failure frequently ensues within months, with many requiring ventilatory support in the first year after onset. Overall survival is markedly reduced compared with other ALS subtypes, with life expectancy often less than two years from symptom onset. Despite heterogeneity across FUS gene-associated ALS, the FUS P525L mutation consistently produces one of the earliest-onset and most fulminant clinical courses [5].

Juvenile-onset ALS, defined by symptom onset before age 25, is a rare form of motor neuron disease that is often genetically determined, with distinct genotype-phenotype correlations guiding diagnosis and prognosis. In addition to FUS gene mutations, several other genes have been implicated. ALS2 mutations typically cause a slowly progressive, upper motor neuron-predominant phenotype with spasticity, dysarthria, dysphagia, and bulbar weakness. SETX mutations often present with distal leg weakness accompanied by cerebellar signs. SPG11 mutations usually manifest with combined upper and lower motor neuron involvement, distal extremity weakness, and early bulbar symptoms [14]. Differentiating among these mutations is crucial, as the age of onset, rate of progression, pattern of motor involvement, and prognosis vary significantly. Early recognition can inform clinical monitoring and consideration for mutation-specific therapeutic trials.

Treatment for juvenile-onset, bulbar-predominant ALS associated with FUS gene mutations remains largely supportive [7]. Riluzole and, when tolerated, edaravone are offered early, although their benefits are modest and not mutation-specific. Aggressive bulbar involvement typically necessitates rapid escalation to gastrostomy for nutrition, followed by non-invasive ventilation and often early tracheostomy. Early engagement of a multidisciplinary ALS care team is essential to preserve communication, mobility, and quality of life. One promising treatment for FUS gene-associated ALS, including the FUS P525L mutation, is the antisense drug ION363, which reduces harmful FUS protein levels and is currently being tested in a major Phase 3 trial following positive early results [7,15]. For now, prompt genetic diagnosis, anticipatory respiratory and nutritional support, and comprehensive palliative care remain the best strategies for managing this relentlessly progressive phenotype.

Early diagnosis is crucial in juvenile-onset, bulbar-predominant ALS because timely identification of the disease allows for earlier intervention that can significantly impact patient outcomes. In this form of ALS, symptoms often progress rapidly, leading to severe bulbar dysfunction that compromises swallowing and respiratory function [5]. Delayed recognition, as seen in our patient, can result in profound malnutrition, increased risk of respiratory infections like pneumonia, seizure provocation due to metabolic disturbances, and the need for invasive interventions such as PEG feeding tubes and tracheostomy. Early diagnosis enables prompt initiation of nutritional support and respiratory care, which may reduce complications, improve quality of life, and potentially extend survival. Moreover, identifying the disease early allows for timely genetic counseling and access to emerging therapies or clinical trials. Therefore, heightened clinical awareness and rapid diagnostic evaluation are essential to optimize management in this aggressive and debilitating condition.

Genetic counseling is essential for patients diagnosed with hereditary ALS, particularly given the autosomal dominant inheritance pattern seen in many FUS gene-associated cases [5]. Although the patient had no formal family history of ALS, his biological mother reportedly experienced similar bulbar symptoms at a young age, suggesting a likely inherited mutation.

In summary, this case underscores the importance of considering juvenile-onset ALS in the differential diagnosis of rapidly progressive bulbar dysfunction in young adults. The identification of an FUS P525L mutation not only confirms the diagnosis but also carries important prognostic implications. Early diagnosis and comprehensive supportive care remain the cornerstones of management.

## Conclusions

This case illustrates the critical importance of considering juvenile-onset ALS in the differential diagnosis of rapidly progressive bulbar symptoms in young adults, particularly when initial evaluations for infectious, structural, and autoimmune causes are unrevealing. Isolated dysphagia and dysphonia of unknown etiology can obscure early recognition of motor neuron disease, leading to delayed diagnosis and management. The identification of a pathogenic FUS P525L mutation in our patient not only confirmed the diagnosis but also provided valuable prognostic information, given the mutation’s association with a particularly aggressive disease course. Early recognition, timely initiation of supportive care, and referral for genetic counseling are essential to improving quality of life and informing future care in patients with hereditary ALS.
